# Leaf elemental composition analysis in spider plant [*Gynandropsis gynandra* L. (Briq.)] differentiates three nutritional groups

**DOI:** 10.3389/fpls.2022.841226

**Published:** 2022-09-02

**Authors:** Aristide Carlos Houdegbe, Enoch G. Achigan-Dako, E. O. Dêêdi Sogbohossou, M. Eric Schranz, Alfred O. Odindo, Julia Sibiya

**Affiliations:** ^1^Discipline of Plant Breeding, School of Agricultural, Earth and Environmental Sciences, University of KwaZulu-Natal, Pietermaritzburg, South Africa; ^2^Laboratory of Genetics, Biotechnology and Seed Science, Faculty of Agronomic Sciences, University of Abomey-Calavi, Abomey-Calavi, Benin; ^3^Biosystematics Group, Wageningen University, Wageningen, Netherlands; ^4^Discipline of Crop Science, School of Agricultural, Earth and Environmental Sciences, University of KwaZulu-Natal, Pietermaritzburg, South Africa

**Keywords:** African leafy vegetable, breeding, *Cleome gynandra*, genetic diversity, human nutrition, local adaptation, nutrient content

## Abstract

Understanding the genetic variability within a plant species is paramount in implementing a successful breeding program. Spider plant (*Gynandropsis gynandra*) is an orphan leafy vegetable and an extraordinary source of vitamins, secondary metabolites and minerals, representing an important resource for combatting malnutrition. However, an evaluation of the leaf elemental composition, using a worldwide germplasm collection to inform breeding programs and the species valorization in human nutrition is still lacking. The present study aimed to profile the leaf elemental composition of *G. gynandra* and depict any potential geographical signature using a collection of 70 advanced lines derived from accessions originating from Asia and Eastern, Southern and West Africa. The collection was grown in a greenhouse using a 9 × 8 alpha lattice design with two replications in 2020 and 2021. Inductively coupled plasma–optical emission spectrometry was used to profile nine minerals contents. A significant difference (*p* < 0.05) was observed among the lines for all nine minerals. Microelements such as iron, zinc, copper and manganese contents ranged from 12.59–430.72, 16.98–166.58, 19.04–955.71, 5.39–25.10 mg kg^−1^ dry weight, respectively, while the concentrations of macroelements such as potassium, calcium, phosphorus and magnesium varied in the ranges of 9992.27–49854.23, 8252.80–33681.21, 3633.55–14216.16, 2068.03–12475.60 mg kg^−1^ dry weight, respectively. Significant and positive correlations were observed between iron and zinc and calcium and magnesium. Zinc, calcium, phosphorus, copper, magnesium, and manganese represented landmark elements in the genotypes. Eastern and Southern African genotypes were clustered together in group 1 with higher phosphorus, copper and zinc contents than Asian and West African lines, which clustered in group 2 and were characterized by higher calcium, magnesium and manganese contents. An additional outstanding group 3 of six genotypes was identified with high iron, zinc, magnesium, manganese and calcium contents and potential candidates for cultivar release. The genotype × year interaction variance was greater than the genotypic variance, which might translate to phenotypic plasticity in the species. Broad-sense heritability ranged from low to high and was element-specific. The present results reveal the leaf minerals diversity in spider plant and represent a baseline for implementing a minerals-based breeding program for human nutrition.

## Introduction

Understanding the plant elemental composition is crucial for both plants and humans. The elemental composition relates to any share of the ionome; this latter refers to the composition of mineral nutrients and trace elements of an organism and constitutes the inorganic component of cellular and organismal systems, such as the leaf, seed or whole plant (Salt et al., 2008). The minerals include macroelements such as carbon (C), nitrogen (N), potassium (K), oxygen (O), calcium (Ca), magnesium (Mg), phosphorus (P), hydrogen (H), and sulfur (S); microelements such as copper (Cu), zinc (Zn), manganese (Mn), iron (Fe), molybdenum (Mo), boron (B), nickel (Ni), and chlorine (Cl), which are essential for plants; and beneficial elements such as cobalt (Co), aluminum (Al), sodium (Na), selenium (Se) and silicon (Si) (Pilon-Smits et al., 2009; Kirkby, 2012). In plants, macro- and micro-elements are key components of biochemical and physiological processes, including DNA synthesis, photosynthesis, chlorophyll biosynthesis, protein modifications, nitrogen fixation and sugar metabolism (Hänsch and Mendel, 2009; Maathuis, 2009; Singh et al., 2016). The plant elemental composition is an essential component of human nutrition, particularly in the current situation where mineral deficiencies or hidden hunger affect more than two billion people worldwide, with the majority living in low- and middle-income countries, mainly in Asia and sub-Saharan Africa (Tulchinsky, 2010; FAO, IFAD, UNICEF, WFP, and WHO, 2019). Elemental composition profiling is often conducted using high-throughput technologies, including inductively coupled plasma–atomic emission spectrometry (ICP–AES), inductively coupled plasma–mass spectrometry (ICP–MS), X-ray fluorescence (XRF) and neutron activation analysis (NAA) (Salt et al., 2008; Huang and Salt, 2016).

The plant elemental composition results from the complex interaction among minerals and is controlled by genetic and physiological processes, although it is also affected by the environment (Baxter, 2009). The main environmental factor driving the changes or variations in plant elemental composition is the soil because almost all the required mineral nutrients and trace elements are absorbed from the soil. For instance, Hogan et al. (2021) observed changes in the root and leaf tissue elemental composition of plant species across a fertility gradient. The plant mineral composition can inform environmental or ecological adaptation. For instance, leaf elemental composition profiling discriminated accessions from different European ecological regions in *Arabidopsis halleri* (L.) O’Kane and Al-Shehbaz (Stein et al., 2017), and the fruit elemental composition revealed geographical signatures in Indian accessions of *Artocarpus heterophyllus* Lam. (Debbarma et al., 2021). The elemental composition is species-specific (White et al., 2012; Watanabe et al., 2016; Neugebauer et al., 2020) but mainly driven by phylogeny (Zhang et al., 2021a) and life forms (Watanabe and Azuma, 2021). In addition, the elemental composition is tissue-specific (Watanabe et al., 2016; Neugebauer et al., 2020) and cultivar-specific (Watanabe et al., 2016; Coulibali et al., 2020) and depends on the growth stage (Huang and Salt, 2016). Thus, genotype × environment interaction is a driving force of elemental accumulation in particular plant species/crop. Understanding the natural variation of the elemental composition among landraces, varieties, or genotypes will provide insights into their adaptation to various local environments of occurrence and provide a solid basis for minerals-based breeding programs to tackle hidden hunger.

Vegetables, particularly orphan or underutilized vegetables, are an important source of micronutrients and represent an affordable source of minerals for local communities (Mwadzingeni et al., 2021; Sarker et al., 2022). The increasing interest in orphan leafy vegetables is particularly due to their distinct richness in minerals, vitamins, phytochemicals and antioxidants (Orech et al., 2007; Nyadanu and Lowor 2015; Moyo et al., 2018) and good adaptation to local conditions. They represent a good asset to adapt to environmental constraints such as drought (Sarker and Oba, 2018) and salinity (Sarker et al., 2018; Hossain et al., 2022). Some of the most nutritious orphan African leafy vegetables include amaranth (*Amaranthus* spp.), spider plant [*Gynandropsis gynandra* (L.) Briq.], African nightshade (*Solanum* spp.), celosia (*Celosia argentea* L.), gboma eggplant (*Solanum macrocarpon* L.), jew’s mallow (*Corchorus olitorius* L.) and Ethiopian kale (*Brassica carinata* A. Braun) (Grubben et al., 2014).

Spider plant (*G. gynandra* syn. *Cleome gynandra* L.), belonging to the Cleomaceae family, is an annual herb with increasing interest because of its high vitamin, mineral and secondary metabolite contents, representing an important resource for combatting malnutrition (Schӧnfeldt and Pretorius, 2011; Neugart et al., 2017; Omondi et al., 2017; Moyo et al., 2018; Gowele et al., 2019; Sogbohossou et al., 2019, 2020; Chataika et al., 2021; Thovhogi et al., 2021). The reported minerals in leaves, which are the most consumed parts of spider plant, include iron, zinc, calcium, copper, potassium, magnesium, manganese, phosphorus and sodium (Jiménez-Aguilar and Grusak, 2015; Omondi et al., 2017; Gowele et al., 2019; Thovhogi et al., 2021). Most previous studies assessing elemental composition in leaves of *G. gynandra* aimed at revealing its superiority in macro- and microelements over popular vegetables for human nutrition. Furthermore, these studies used various methods and technologies, and germplasm was limited to a country or region in Africa, with the most prominent being the study of Omondi et al. (2017). Although significant variation was observed, these authors did not attempt to identify landmark elements to differentiate accessions in the species, as has been shown in the species based on morphology (Wu et al., 2018; Sogbohossou et al., 2019) and secondary metabolites (Sogbohossou et al., 2020) using worldwide accessions. Therefore, knowledge of natural elemental composition variation among geographically diverse accessions of *G. gynandra* is limited.

We hypothesized that: (i) there is a significant variation in leaf mineral variation in a worldwide assembled genotypes of *G. gynandra* from Asia and Eastern, Southern and West Africa; (ii) the leaf elemental composition of spider plant’s genotypes depends on the geographical origin of genotype; (iii) the leaf mineral elements content in *G. gynandra* are interrelated to guide direct selection; and (iv) leaf minerals content is moderately heritable in *G. gynandra*. The objectives of the present study were to: (i) profile the leaf elemental composition of *G. gynandra* using worldwide assembled genotypes from Asia and Eastern, Southern and West Africa; (ii) determine the potential geographical signature of the leaf elemental composition; (iii) assess the relationship among the mineral element concentrations; and (iv) estimate the quantitative genetic parameters of element composition in the leaves of *G. gynandra*.

## Materials and methods

### Plant material

Seventy advanced lines developed from 70 accessions (Table 1) originating from Asia (18), West Africa (18), Eastern Africa (14) and Southern Africa (20) were used in this study. The initial accessions were obtained from the germplasm collection of the Laboratory of Genetics, Biotechnology and Seed Science of the University of Abomey-Calavi (Republic of Benin); the World Vegetable Center (Taiwan); the Kenya Resource Center for Indigenous Knowledge (Kenya); the Lilongwe University of Agriculture and Natural Resources (Malawi); the Namibia Botanical Gardens (Namibia); the Wageningen University and Research (Netherlands) and the University of Ouagadougou (Burkina-Faso). The advanced lines were developed through four generations of self-pollination.

**Table 1:** List of advanced lines of *Gynandropsis gynandra* used in this study and their origin.

Genotype	Genebank of the original accession	Country of origin	Generation of selfing	Region
EA1	National Museums of Kenya	Kenya	S4	Eastern Africa
EA2	National Museums of Kenya	Kenya	S4	Eastern Africa
EA3	National Museums of Kenya	Kenya	S4	Eastern Africa
EA4	National Museums of Kenya	Kenya	S4	Eastern Africa
WA1	University of Ouagadougou	Burkina-Faso	S4	West Africa
WA2	University of Ouagadougou	Burkina-Faso	S4	West Africa
EA5	National Museums of Kenya	Kenya	S4	Eastern Africa
EA6	National Museums of Kenya	Kenya	S4	Eastern Africa
WA3	University of Ouagadougou	Burkina-Faso	S4	West Africa
WA4	Laboratory of Genetics, Biotechnology and Seed Science (GBioS), University of Abomey-Calavi	Benin	S4	West Africa
WA5	Laboratory of Genetics, Biotechnology and Seed Science (GBioS), University of Abomey-Calavi	Benin	S4	West Africa
WA6	Laboratory of Genetics, Biotechnology and Seed Science (GBioS), University of Abomey-Calavi	Benin	S4	West Africa
WA7	Laboratory of Genetics, Biotechnology and Seed Science (GBioS), University of Abomey-Calavi	Benin	S4	West Africa
WA8	Laboratory of Genetics, Biotechnology and Seed Science (GBioS), University of Abomey-Calavi	Benin	S4	West Africa
WA9	Laboratory of Genetics, Biotechnology and Seed Science (GBioS), University of Abomey-Calavi	Benin	S4	West Africa
WA10	Laboratory of Genetics, Biotechnology and Seed Science (GBioS), University of Abomey-Calavi	Benin	S4	West Africa
WA11	Laboratory of Genetics, Biotechnology and Seed Science (GBioS), University of Abomey-Calavi	Togo	S4	West Africa
WA12	Laboratory of Genetics, Biotechnology and Seed Science (GBioS), University of Abomey-Calavi	Togo	S4	West Africa
WA13	Laboratory of Genetics, Biotechnology and Seed Science (GBioS), University of Abomey-Calavi	Togo	S4	West Africa
WA14	Laboratory of Genetics, Biotechnology and Seed Science (GBioS), University of Abomey-Calavi	Togo	S4	West Africa
WA15	Laboratory of Genetics, Biotechnology and Seed Science (GBioS), University of Abomey-Calavi	Togo	S4	West Africa
WA16	Laboratory of Genetics, Biotechnology and Seed Science (GBioS), University of Abomey-Calavi	Togo	S4	West Africa
WA17	Laboratory of Genetics, Biotechnology and Seed Science (GBioS), University of Abomey-Calavi	Togo	S4	West Africa
WA18	Laboratory of Genetics, Biotechnology and Seed Science (GBioS), University of Abomey-Calavi	Togo	S4	West Africa
AS1	World Vegetable Center	Thailand	S4	Asia
AS2	World Vegetable Center	Lao People’s Democratic Republic	S4	Asia
AS3	World Vegetable Center	Lao People’s Democratic Republic	S4	Asia
AS4	World Vegetable Center	Lao People’s Democratic Republic	S4	Asia
AS5	World Vegetable Center	Thailand	S4	Asia
AS6	World Vegetable Center	Thailand	S4	Asia
EA7	World Vegetable Center	Kenya	S4	Eastern Africa
SA1	World Vegetable Center	Zambia	S4	Southern Africa
AS7	World Vegetable Center	Lao People’s Democratic Republic	S4	Asia
AS8	World Vegetable Center	Malaysia	S4	Asia
AS9	World Vegetable Center	Malaysia	S4	Asia
AS10	World Vegetable Center	Malaysia	S4	Asia
AS11	World Vegetable Center	Malaysia	S4	Asia
AS12	World Vegetable Center	Lao People’s Democratic Republic	S4	Asia
EA8	World Vegetable Center	Uganda	S4	Eastern Africa
EA9	World Vegetable Center	Uganda	S4	Eastern Africa
EA10	World Vegetable Center	Uganda	S4	Eastern Africa
EA11	World Vegetable Center	Uganda	S4	Eastern Africa
SA2	World Vegetable Center	Malawi	S4	Southern Africa
SA3	World Vegetable Center	Malawi	S4	Southern Africa
EA12	World Vegetable Center	Kenya	S4	Eastern Africa
EA13	World Vegetable Center	Kenya	S4	Eastern Africa
SA4	World Vegetable Center	South Africa	S4	Southern Africa
SA5	World Vegetable Center	Zambia	S4	Southern Africa
AS13	World Vegetable Center	Taiwan	S4	Asia
SA6*	Laboratory of Genetics, Biotechnology and Seed Science (GBioS), University of Abomey-Calavi	Mozambique	S4	Southern Africa
EA14	National Museums of Kenya	Kenya	S4	Eastern Africa
AS14	World Vegetable Center	Malaysia	S4	Asia
AS15	World Vegetable Center	Thailand	S4	Asia
AS16	World Vegetable Center	Lao People’s Democratic Republic	S4	Asia
AS17	World Vegetable Center	Lao People’s Democratic Republic	S4	Asia
SA7	Okakarara	Namibia	S4	Southern Africa
SA8	Otjiwarongo	Namibia	S4	Southern Africa
SA9	Lilongwe University of Agriculture and Natural Resources	Malawi	S4	Southern Africa
SA10	Lilongwe University of Agriculture and Natural Resources	Malawi	S4	Southern Africa
SA11	Mahenene Research Station	Namibia	S4	Southern Africa
SA12	Chitedze Research Station	Malawi	S4	Southern Africa
SA13	Namibia Botanical Gardens	Namibia	S4	Southern Africa
SA14	Namibia Botanical Gardens	Namibia	S4	Southern Africa
SA16*	Laboratory of Genetics, Biotechnology and Seed Science (GBioS), University of Abomey-Calavi	Zimbabwe	S4	Southern Africa
AS18	Wageningen University and Research	Malaysia	S4	Asia
SA17	Okakarara	Namibia	S4	Southern Africa
SA18	Lilongwe University of Agriculture and Natural Resources	Malawi	S4	Southern Africa
SA19	Lilongwe University of Agriculture and Natural Resources	Malawi	S4	Southern Africa
SA20	Chitedze Research Station	Malawi	S4	Southern Africa
SA21*	Laboratory of Genetics, Biotechnology and Seed Science (GBioS), University of Abomey-Calavi	Zimbabwe	S4	Southern Africa

* Provided to the Laboratory of Genetics, Biotechnology and Seed Science (GBioS) of University of Abomey-Calavi by Tomas Massingue (Mozambique) and Admire Shayanowako (Zimbabwe).

### Experimental design and growth conditions

The germplasm was evaluated over two years, from September to December 2020, and from January to April 2021. Both experiments were laid out in a 9 × 8 alpha design with two replicates in a greenhouse at the Controlled Environment Facility (29°46′ S, 30°58′ E) of the University of KwaZulu-Natal, Pietermaritzburg Campus, South Africa. Seeds were pretreated by heating at 40°C for 3 days to improve germination before sowing in cell trays filled with growing media. Cell trays were established in the greenhouse. Seedlings were grown for four weeks in a nursery and transplanted in 14 l (27 cm height × 30 cm top diameter × 21 cm bottom diameter) tapered cylindrical pots with three plants per pot. Pots were filled with composted pine bark growing media. The growing media was characterized by 30.78% of carbon, 1.10% of nitrogen, 1.35% of calcium, 0.33% of magnesium, 0.25% of potassium, 0.34% of phosphorus, 469.99 mg kg^−1^ of sodium, 181.35 mg kg^−1^ of zinc, 42.41 mg kg^−1^ of copper, 1034.08 mg kg^−1^ of manganese, 13349.42 mg kg^−1^ of iron, and 6452.14 mg kg^−1^ of aluminum on dry matter basis. Basal fertilizer composed of N:P:K (2:3:2) at a dose of 150 kg ha^−1^ was applied before transplanting, and limestone ammonium nitrate (28% N) was applied as topdressing two weeks after transplanting at a dose of 100 kg ha^−1^. Automated drip irrigation was used to water the plants, while weeds were controlled manually. During 2020, the average temperature and relative humidity were 28°C day/20°C night and 78.5%, respectively. In 2021, the average temperature and relative humidity were 31° C day/22°C night and 77.4%, respectively.

### Minerals analysis

Four weeks after transplanting, leaves were randomly collected in paper bags from all the plants in each replicate and bulked to obtain at least 20 g per genotype. The collected leaves were immediately transported to the laboratory, washed and oven-dried at 65°C for 72 h. After cooling, dried leaves were ground using a mortar and pestle into a powder and sieved using a 1 mm screen sieve. Two independent replicates of 0.5 g each of sieved powder were weighed in porcelain crucibles using an analytical balance (D & T, ES-E200A, max = 200 g, d = 0.1 mg, China). Samples were afterwards ashed in a muffle furnace at 550°C for 2 h. The obtained ashes were digested using 10 ml of double acid composed of nitric acid (HNO_3_, 65%, Merck, Germany) and hydrochloric acid (HCl, 32%, Merck, Germany) mixed at a ratio of 1:3 (Jones, 2001). The resultant mixtures were placed on a hot plate at 250°C for 30 min and later cooled for 1 h. Digestates were filtered using Whatman paper Grade 1 (Qualitative Filter Paper Standard Grade, circle, 125 mm, Merck, Germany) into a 100 ml volumetric flask and made up to the mark using deionized water. The resultant solutions were analyzed using an inductively coupled plasma-optical emission spectrometer (ICP–OES; Varian 720-ES, Varian Inc., Mulgrave, Victoria, Australia) for Ca, Cu, Fe, K, Mn, Mg, Na, P and Zn at the ICP Laboratory of the School of Chemistry and Physics of the University of KwaZulu-Natal, Pietermaritzburg Campus. The wavelengths used were 317.933 nm for Ca, 324.754 nm for Cu, 259.940 nm for Fe, 766.491 nm for K, 279.078 nm for Mg, 257.610 nm for Mn, 588.995 nm for Na, 213.618 nm for P, and 213.857 nm for Zn. An ICP multielement aqueous certified reference standard (1,000 μg mL^−1^ ULTRASPEC^®^ 5% HNO_3_) was purchased from De Bruyn Spectroscopic Solutions Company, South Africa and used for calibration. All mineral contents were reported in mg kg^−1^ on a dry weight basis (mg kg^−1^ DW).

### Data analysis

All statistical analyses were conducted in R software version 4.1.1 (R Core Team, 2021). Data quality was assessed for outlier detection according to Bernal-Vasquez et al. (2016) using the Bonferroni–Holm test based on studentized residuals at the level of significance of 5%. The normality of the data was assessed using the Shapiro Wilk test, and only magnesium data were normally distributed. Descriptive statistics, including mean, minimum, maximum, range, coefficient of variation, and standard error, were generated to characterize the germplasm using the function *describe()* from the R package “psych” (Revelle, 2019). When necessary, the difference among genotypes and regions of origin was tested through an analysis of variance or Kruskal–Wallis test using the function *aov()* or *kruskal.test()*, respectively. Variance components for each mineral were estimated in each year and across years. Each year, data were analyzed separately by implementing a linear mixed model following this statistical model:


(1) $$y_{ik}=\mu +R_{k}+G_{i}+\varepsilon_{ik}$$

in which $y_{ik}$ was the phenotypic observation of the *i^th^* genotype in the *k^th^* replicate, μ was the overall mean, $R_{k}$ was the random effect of the *k^th^* replicate, $G_{i}$ was the random effect of the *i^th^* genotype, and $\varepsilon_{ik}$ was the random residual. Broad-sense heritability was calculated according to Hallauer et al. (2010) as follows:


(2) $$H^{2}=\sigma_{G}^{2}/\left(\sigma_{G}^{2}+\sigma_{e}^{2}/r\right)$$

where $\sigma_{G}^{2}$ is the total genotypic variance, $\sigma_{e}^{2}$ is the residual variance, and r is the number of replications.

Variance components across years were estimated by fitting a linear mixed-effect model according to the following statistical model:


(3) $$y_{ijk}=\mu +Y_{j}+R_{k}\left(Y_{j}\right)+G_{i}+GY_{ij}+\varepsilon_{ijk}$$

in which $y_{ijk}$ was the phenotypic observation of the *i^th^* genotype in the *k^th^* replicate at the *j^th^* year, μ was the overall mean, $Y_{j}$ was the random effect of the *j^th^* year, $R_{k}\left(Y_{j}\right)$ was the random effect of the *k^th^* replicate within the *j^th^* year, $G_{i}$ was the random effect of the *i^th^* genotype, $GY_{ij}$ was the random effect of the interaction between the *i^th^* genotype and the *j^th^* year, and $\varepsilon_{ijk}$ was the random residual. Residual variances were assumed to be heterogeneous among years. Broad-sense heritability across years was calculated according to Hallauer et al. (2010) as follows:


(4) $$H^{2}=\sigma_{G}^{2}/\left(\sigma_{G}^{2}+\sigma_{G\times Y}^{2}/n+\sigma_{e}^{2}/nr\right)$$

where $\sigma_{G}^{2}$ is the total genotypic variance, $\sigma_{G\times Y}^{2}$ is the genotype × year interaction variance, $\sigma_{e}^{2}$ is the residual variance, *r* is the number of replications, and *n* is the number of years. As adjusted means across years, the best linear unbiased estimators (BLUEs) were estimates from the across years analysis, assuming fixed genotype effects. The BLUEs were therefore used in the further analyses. All linear mixed-effects models were fitted using the restricted maximum likelihood (REML) implemented in the “ASReml-R” package version 4.1.0.160 (Butler et al., 2017). Phenotypic and genotypic correlation coefficients among all leaf mineral elements and their significance level were calculated using META-R software version 6.04 (Alvarado et al., 2020). Path coefficient analysis was carried out using the *path.analysis()* function of the R “agricolae” package (de Mendiburu, 2021) to partition phenotypic correlation into direct and indirect effects of other minerals contents on iron content. To assess the relationship among the genotypes and the leaf mineral elements, a principal component analysis was performed using the *PCA()* function implemented in the R “FactoMineR” package (Lê et al., 2008). Furthermore, we performed a hierarchical clustering on principal components (HCPC) using the *HCPC()* function of the same R package to group the genotypes based on the minerals, and the results were visualized as a factor map using the *fviz_cluster()* function of the R package “factoextra” (Kassambara and Mundt, 2020). Significant differences among clusters were tested using the Kruskal–Wallis test followed by Dunn’s post-hoc test for mean separation using the function *dunn.test()* from the R package “dunn.test” (Dinno, 2017). The genetic advance (GA) for each mineral was computed as:


(5) $$GA=i\times H^{2}\times \sigma_{P}$$

where $\sigma_{P}$ is the phenotypic standard deviation, $H^{2}$ is the broad-sense heritability, and *i* is the standardized selection differential at a selection intensity of 5% (i = 2.06) (Singh and Chaudhary, 1985). Genetic advance over mean (GAM) was further computed as:


(6) $$GAM=\left(GA/\bar{x}\right)\times 100$$

where $\bar{x}$ and GA are the genetic advance and the overall mean of the element content, respectively. Genotypic coefficient of variation (GCV), phenotypic coefficient of variation (PCV) and error coefficient of variation (ECV) were estimated as described by Burton and DeVane (1953) as follows:


(7) $$GCV\;\left(\%\right)=\frac{\sqrt{\sigma_{G}^{2}}}{\bar{x}}\times 100$$

(8) $$PCV\;\left(\%\right)=\frac{\sqrt{\sigma_{P}^{2}}}{\bar{x}}\times 100$$

(9) $$ECV\;\left(\%\right)=\frac{\sqrt{\sigma_{e}^{2}}}{\bar{x}}\times 100$$

where $\sigma_{G}^{2}$ is the genotypic variance, $\sigma_{P}^{2}$ is the phenotypic variance, $\sigma_{e}^{2}$ is the residual variance, and $\bar{x}$ is the overall mean.

## Results

### Macroelements profile of leaves in *Gynandropsis gynandra*

**Figure 1:**
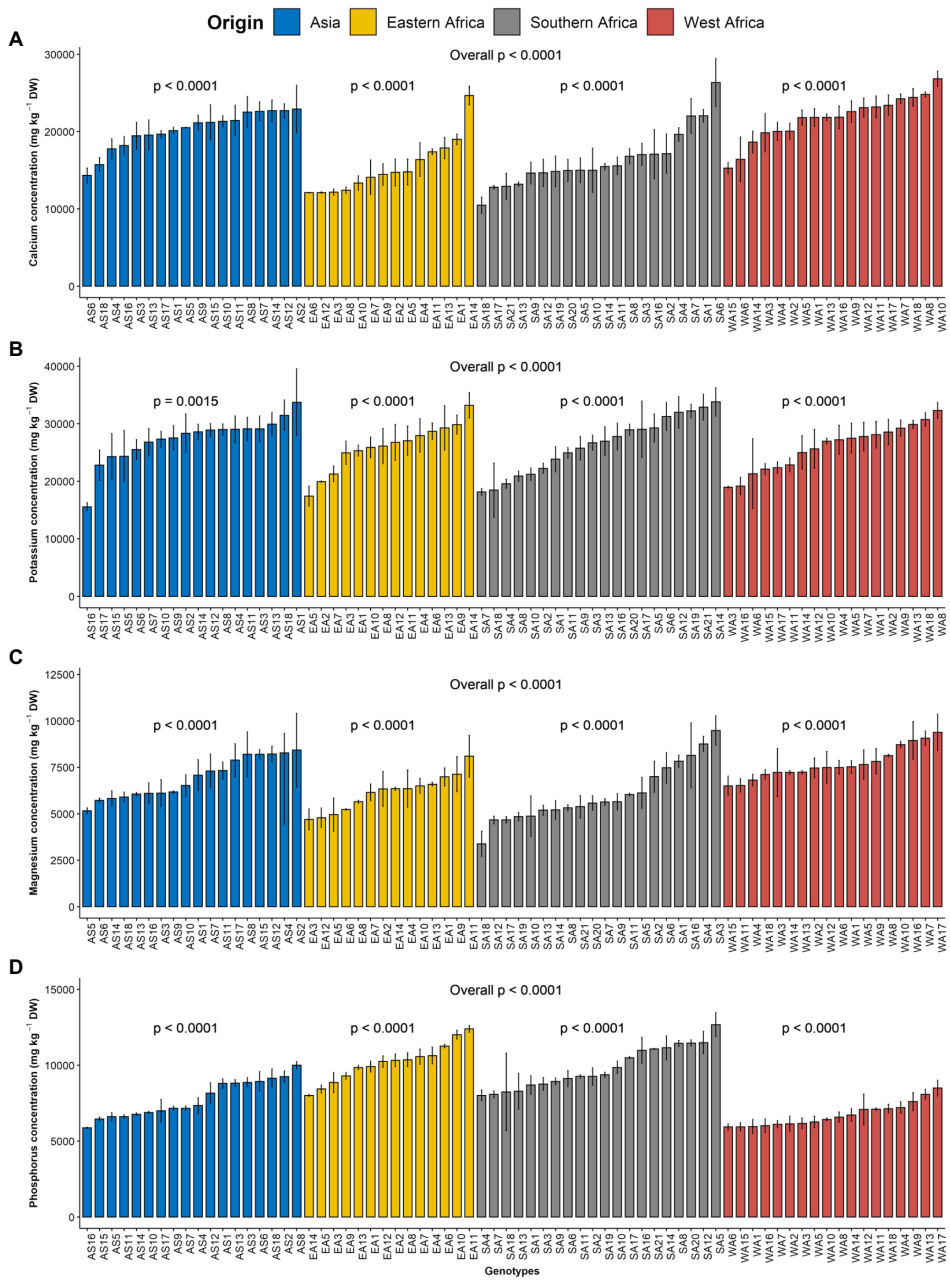
Variation in leaf macroelements content among 70 advanced lines of *Gynandropsis gynandra* evaluated across years (2020 and 2021). **(A)** Calcium content; **(B)** Potassium content; **(C)** Magnesium content and **(D)** Phosphorus content. Bar plots are means and error bars represent standard errors across years (*n* = 4).

The macroelements detected at significant levels in the leaves of *G. gynandra* included calcium, potassium, phosphorus and magnesium (Table 2). The most abundant macroelement was potassium, with content ranging from 9992.27 to 49854.23 with a mean of 26393.85 mg kg^−1^ dry weight (DW), followed by calcium (8252.8–33681.21 mg kg^−1^ DW), phosphorus (3633.55–14216.16 mg kg^−1^ DW) and magnesium (2068.03–12475.6 mg kg^−1^ DW), with average contents of 18539.7, 8558.29 and 6719.83 mg kg^−1^ DW, respectively. A highly significant difference (*p* < 0.001) was observed among lines overall and within the region of origin for all macroelements contents (Figure 1). In addition, regions of origin differed significantly (*p* < 0.001) for all macroelements contents except for potassium content (Supplementary Figures 1A–D). On average, lines originating from West Africa had the highest calcium and magnesium contents, followed by the Asian lines. In contrast, Eastern and Southern African genotypes had the highest phosphorus content, whereas West African genotypes had the lowest phosphorus content (Supplementary Figures 1A–D).

**Table 2:** Descriptive statistics of nine mineral contents in a population of 70 advanced lines of *G. gynandra* evaluated across years (2020 and 2021).

Minerals	Mean	Minimum	Maximum	Range	Standard error	Coefficient of variation (%)
Ca	18539.7	8252.8	33681.21	25428.41	287.92	25.94
Cu	12.17	5.39	25.1	19.71	0.23	31.35
Fe	133.04	12.59	430.72	418.14	3.53	43.98
K	26393.85	9992.27	49854.23	39861.96	355.74	22.51
Mg	6719.83	2068.03	12475.6	10407.56	109.61	27.15
Mn	217.68	19.04	955.71	936.67	8.75	66.98
Na	1143.55	535.92	2165.9	1629.98	16.91	24.61
P	8558.29	3633.55	14216.16	10582.61	122.85	23.89
Zn	55.85	16.98	166.58	149.59	1.29	38.67

All minerals concentration (mg kg^−1^ dry weight).

### Microelements profile of leaves in *Gynandropsis gynandra*

**Figure 2:**
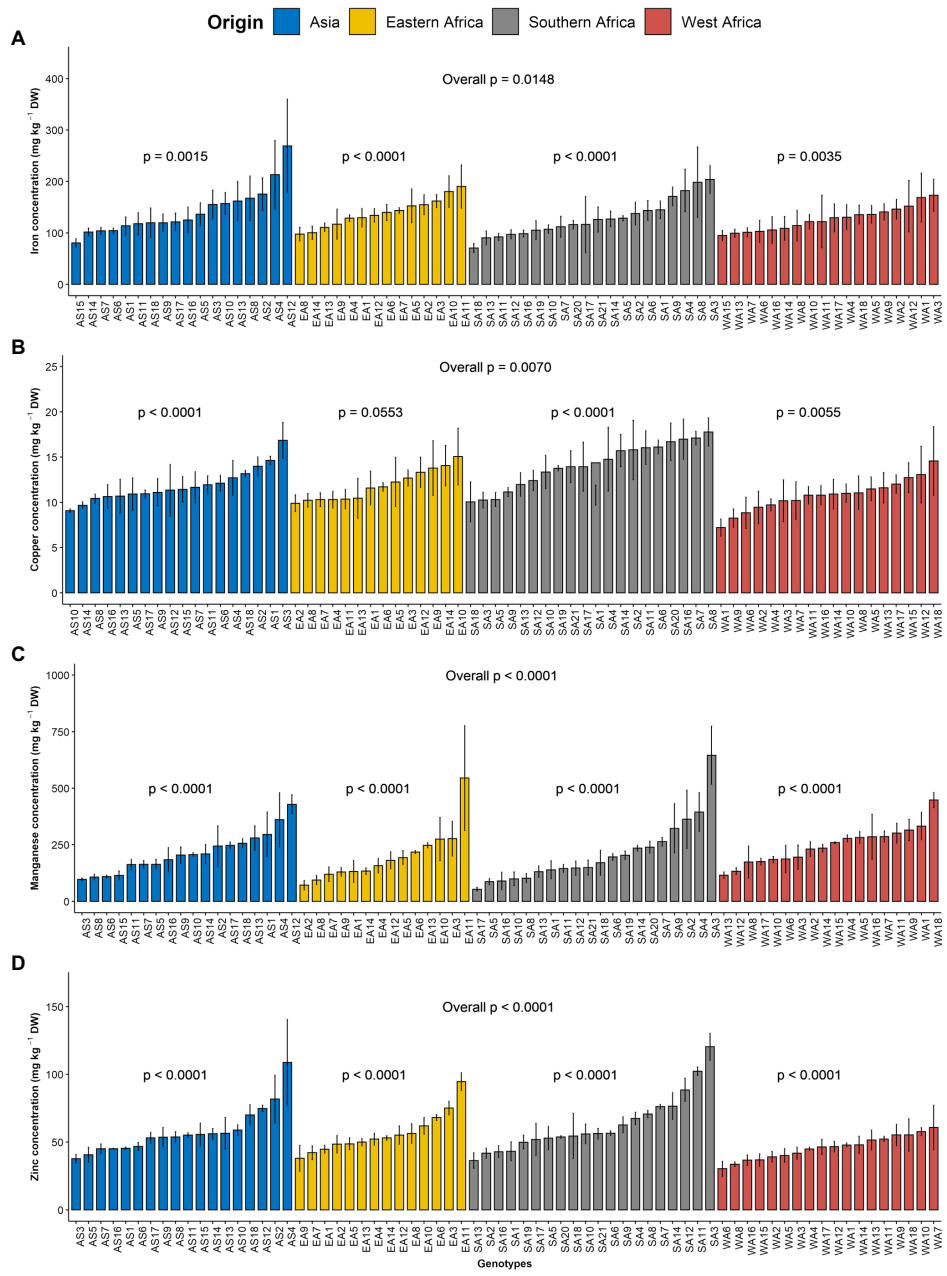
Variation in leaf microelements content among 70 advanced lines of *G. gynandra* evaluated across years (2020 and 2021). **(A)** Iron content; **(B)** Copper content; **(C)** Manganese content and **(D)** Zinc content. Bar plots are means and error bars represent standard errors across years (*n* = 4).

The order of importance of microelements contents in the leaves of *G. gynandra* was manganese > iron > zinc > copper. The manganese content varied from 19.04 to 955.71 mg kg^−1^ DW, with the highest variability (CV = 66.98%). The iron content ranged between 12.59 and 430.72 with an average of 133.04 mg kg^−1^ DW and constituted the second most variable microelement in the leaf. The zinc content had a CV of 38.67% and varied between 16.98 and 166.58, with an average of 55.85 mg kg^−1^ DW. With the lowest CV (31.35%), the copper content was 12.17 mg kg^−1^ DW on average with a range of 5.39–25.1 mg kg^−1^ DW. For iron, a significant difference (*p* < 0.05) was noticed among genotypes overall, while a high significant difference (*p* < 0.01) was observed among genotypes within each region (Figure 2A). Although no significant difference (*p* = 0.162) was observed among regions of origin across the 2 years for iron content, a significant difference (*p* < 0.05) was observed among regions of origin in each year with fluctuating performance of the regions of origin from one year to another (Supplementary Figure 1E). Regarding copper, a very significant difference (*p* < 0.01) was observed among genotypes overall and within Asian, West and Southern African regions, but a marginal level of significance (*p* = 0.055) was observed among Eastern African genotypes (Figure 2B). A highly significant difference (*p* < 0.001) was observed among regions of origin across the two years for copper content, with Southern African genotypes having the highest copper content and West African genotypes having the lowest copper content (Supplementary Figure 1F). Manganese and zinc showed a highly significant difference among genotypes overall and within the region of origin (*p* < 0.001; Figures 2C,D) but also among regions of origin (Supplementary Figures 1G,H). West African genotypes had the highest manganese content, followed by the Asian genotypes and the Eastern and Southern African genotypes (Supplementary Figure 1G). In contrast, the Southern African genotypes had the highest zinc content, while the lowest was observed for West African genotypes (Supplementary Figure 1H).

### Sodium content in leaves of *Gynandropsis gynandra*

**Figure 3:**
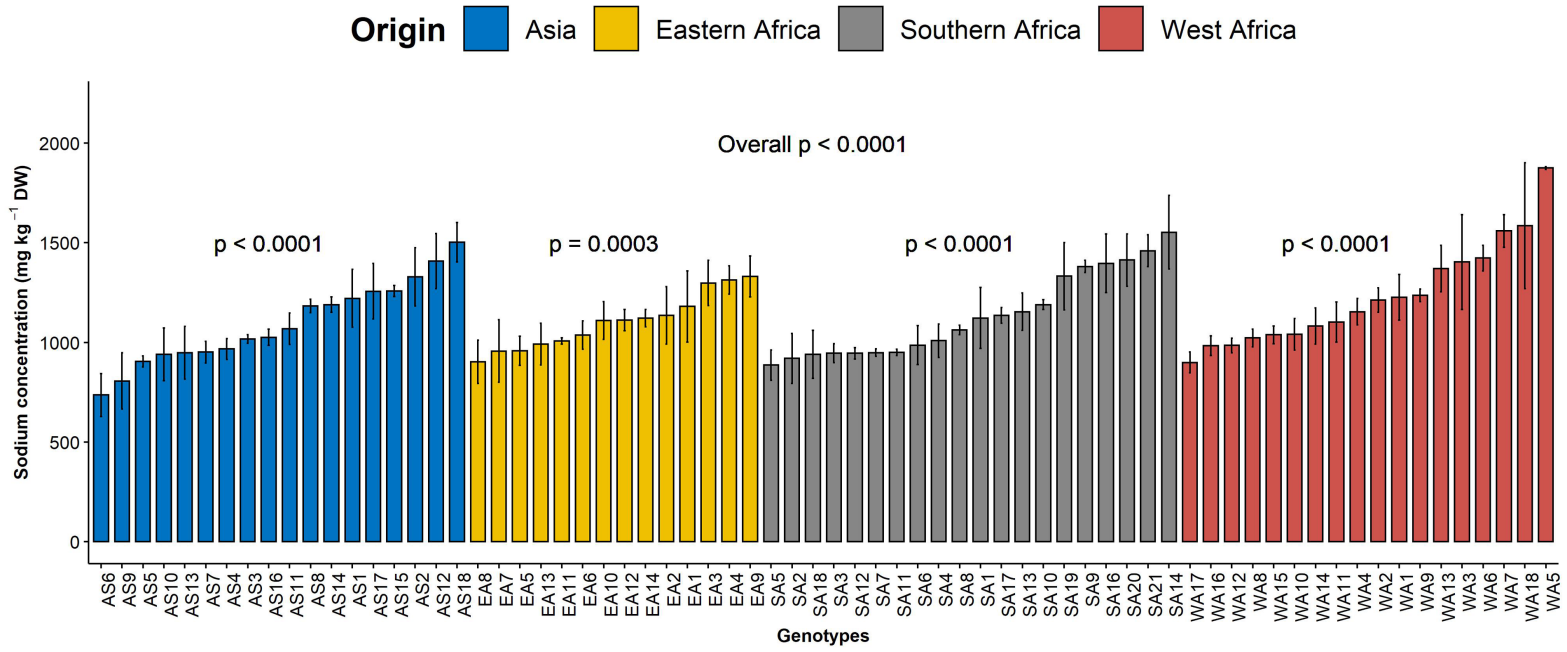
Variation in leaf sodium content among 70 advanced lines of *G. gynandra* evaluated in 2020 and 2021. Bar plots are the means, and error bars represent standard errors across years (*n* = 4).

Sodium is another beneficial element investigated in the present study. The average sodium content was 1143.55 mg kg^−1^ DW with a coefficient of variation of 24.61%. A highly significant difference was noticed among genotypes overall (*p* < 0.001, Figure 3). While a highly significant difference was observed among genotypes within each region for sodium content (Figure 3), no significant difference (*p* = 0.17) was depicted among the regions of origin (Supplementary Figure 1I).

### Estimates of genetic parameters of leaf mineral elements

Estimates of genetic parameters, including variance components, heritability, genetic gain, phenotypic coefficient of variation (PCV), genotypic coefficient of variation (GCV) and error coefficient of variation (ECV), for each element per year and across years are presented in Supplementary Table 1 and Table 3, respectively. Genotypic variance for each leaf elemental composition of *G. gynandra* was higher than the residual variance in each year except for copper and iron in 2021 (Supplementary Table 1). Consequently, high broad-sense heritability was observed and ranged from 0.62 to 0.99 for all mineral contents in both 2020 and 2021 except for copper content (0.41) in 2021. The genotypic and phenotypic coefficients of variation were moderate to high for all mineral contents each year (Supplementary Table 1). Similarly, genetic gains were moderate to high for all element concentrations per year except potassium content (27.92%) in 2020 and copper content (19.20%) in 2021 (Supplementary Table 1). Across years, genotype × year interaction variance was higher than the genotypic variance for all mineral contents except for calcium and phosphorus contents. Broad-sense heritability across years varied from 0.00 to 0.78, with calcium content (0.78 ± 0.05) and phosphorus content (0.76 ± 0.06) having relatively high values. Moderate broad-sense heritability was observed for potassium (0.41 ± 0.14), magnesium (0.56 ± 0.11), manganese (0.31 ± 0.17), sodium (0.35 ± 0.16) and zinc (0.53 ± 0.11) contents. Genotypic variance across years for iron content was null with a heritability equal to zero. Variable genetic gains at 5% selection intensity were observed for the leaf mineral elements with no genetic gain for iron and the highest (35.27% over the mean of the current population) for calcium content (Table 3). The error coefficient of variation was low (<10%) for magnesium, manganese, sodium, phosphorus and zinc contents, moderate for calcium (11.88%) and potassium (14.19%) contents, and high for calcium (22.70%) and iron (31.26%) contents. A high (>20%) coefficient of genotypic variation was noticed for manganese and zinc contents and moderate for calcium, potassium, magnesium, sodium and phosphorus contents (Table 3). The phenotypic coefficient of variation was moderate to high for all leaf mineral elements (Table 3).

**Table 3:** Estimates of genetic parameters for the leaf mineral content in 70 advanced lines of *G. gynandra* evaluated across years (2020 and 2021).

Genetic parameters	Ca	Cu	Fe	K	Mg	Mn	Na	P	Zn
$\sigma_{G}^{2}$	12840936.25 ± 2842625.89	0.73 ± 1.22	0.00 ± NA	7035034.9 ± 3377570.85	987563.75 ± 329479.35	3639.83 ± 2458.75	16228.06 ± 9466.68	2551218.53 ± 587213.33	157.17 ± 55.79
$\sigma_{Y}^{2}$	1842253.44 ± 2749025.01	0.52 ± 1.07	15.81 ± 75.11	127443.83 ± 658427.20	1484647.33 ± 2131238.43	1977.21 ± 3129.67	0.00 ± NA	0.21 ± NA	41.57 ± 64.43
$\sigma_{G\times Y}^{2}$	4664094.80 ± 1238432.51	5.19 ± 1.69	1703.78 ± 330.12	13459157.61 ± 3571965.16	1534003.85 ± 266444.04	16369.1 ± 2807.40	57942.85 ± 10265.64	1547882.61 ± 272119.80	269.59 ± 47.53
$\sigma_{e}^{2}$	4847991.37 ± 813500.07	7.72 ± 1.42	1743.47 ± 293.14	14043558.52 ± 2400108.74	60650.78 ± 10361.10	234.13 ± 40.28	5487.84 ± 930.75	119358.44 ± 20637.24	18.93 ± 3.22
$\sigma_{P}^{2}\;$	16384981.49 ± 2774396.71	5.25 ± 0.92	1287.76 ± 155.95	17275503.34 ± 2912547.10	1769728.37 ± 301346.98	11882.91 ± 2023.28	46571.45 ± 7931.71	3354999.44 ± 571368.64	296.69 ± 50.52
$H^{2}$	0.78 ± 0.05	0.14 ± 0.21	0.00 ± 0.00	0.41 ± 0.14	0.56 ± 0.11	0.31 ± 0.17	0.35 ± 0.16	0.76 ± 0.06	0.53 ± 0.11
Mean	18530.05	12.23	133.57	26413.46	6716.95	216.63	1143.50	8568.63	55.89
GCV (%)	19.34	6.97	0.03	10.04	14.79	27.85	11.14	18.64	22.43
PCV (%)	21.84	18.73	26.87	15.74	19.81	50.32	18.87	21.38	30.82
ECV (%)	11.88	22.70	31.26	14.19	3.67	7.06	6.48	4.03	7.78
GA	6534.93	0.65	0	3486.73	1529.25	68.78	154.91	2869.25	18.80
GAM (%)	35.27	5.34	0	13.2	22.77	31.75	13.55	33.49	33.63

$\sigma_{G}^{2}$, genotypic variance; $\sigma_{Y}^{2}$, year variance; $\sigma_{G\times Y}^{2}$, genotype × year interaction variance; $\sigma_{e}^{2}$, residual variance; $\sigma_{P}^{2}$, phenotypic variance; $H^{2}$, broad-sense heritability; GCV, genotypic coefficient of variation; PCV, phenotypic coefficient of variation; ECV, residual coefficient of variation; GA, genetic advance; GAM, genetic advance over mean.

### Phenotypic and genotypic correlation among leaf elemental components

Phenotypic correlation coefficients (r_p_) and genotypic correlation coefficients (r_g_) between the elemental composition of the leaves in *Gynandropsis gynandra* are summarized in Table 4 and ranged from −0.58 to 0.67 and − 0.99 to 0.82, respectively. The highest, positive, and significant correlation was observed between calcium and magnesium contents (*r_p_* = 0.67, *r_g_* = 0.82, *p* < 0.001), while the highest negative and significant correlation was observed between calcium and phosphorus contents (*r_p_* = −0.58, *p* < 0.001) for phenotypic one and between copper and magnesium (*r_g_* = −0.99, *p* < 0.001), and copper and sodium (*r_g_* = −0.99, *p* < 0.001) contents for genotypic one. Some traits displayed similar patterns for both types of correlations. A moderate, significant, and positive correlation was observed between the concentrations of phosphorus and zinc (*r_p_* = 0.31, *r_g_* = 0.37 *p* < 0.01), magnesium and manganese (*r_p_* = 0.39, *r_g_* = 0.48, *p* < 0.001), and potassium and sodium (*r_p_* = 0.30, *r_g_* = 0.45, *p* < 0.05), and zinc and manganese (*r_p_* = 0.49, *r_g_* = 0.57, *p* < 0.001). While a moderate, significant, and positive correlation was observed between the concentrations of copper and phosphorus (*r_p_* = 0.40, *r_g_* = 0.75, *p* < 0.001), calcium and magnesium (*r_p_* = 0.67, *r_g_* = 0.82, *p* < 0.001) at phenotypic level, a high, significant, and positive correlations were observed at genotypic level between the same elements. Phosphorus content had a moderate, negative, and significant phenotypic correlation, and a high, negative, and significant genotypic correlations with calcium and magnesium contents. There was a moderate and positive phenotypic correlation between iron and manganese (*r_p_* = 0.49, *p* < 0.001) and iron and zinc (*r_p_* = 0.42, *p* < 0.001). At genotypic level, no correlation was found between iron content and all minerals contents. The significant correlation observed between phosphorus and potassium contents (*r_p_* = 0.24, *p* < 0.05) was weak. Copper was not correlated with magnesium, manganese and sodium at phenotypic level but had a highly significant and negative correlation with the same elements at genotypic level (Table 4).

**Table 4:** Phenotypic correlation coefficients (below diagonal) and genotypic correlation coefficients (above diagonal) among nine leaf mineral concentrations in a population of 70 advanced lines of *G. gynandra*.

Minerals	Ca	Cu	Fe	K	Mg	Mn	Na	P	Zn
Ca		−0.20	0.00	0.00	**0.82** ^***^	**0.36** ^**^	−0.02	**−0.79** ^***^	−0.19
Cu	−0.11		0.00	**−0.32** ^**^	**−0.99** ^***^	**−0.79** ^***^	**−0.99** ^***^	**0.75** ^***^	0.12
Fe	0.11	0.01		0.00	0.00	0.00	0.00	0.00	0.00
K	0.13	0.07	−0.02		−0.23	−0.10	**0.45** ^***^	0.23	0.06
Mg	**0.67** ^***^	−0.21	0.28^*^	0.01		**0.48** ^***^	−0.11	**−0.79** ^***^	**−0.36** ^**^
Mn	0.17	−0.16	**0.49** ^***^	0.03	**0.39** ^***^		0.04	**−0.38** ^***^	**0.57** ^***^
Na	0.06	0.03	0.03	**0.30** ^*^	0.11	0.12		−0.19	**−0.45** ^***^
P	**−0.58** ^***^	**0.40** ^***^	0.14	**0.24** ^*^	**−0.38** ^**^	−0.16	−0.06		**0.37** ^**^
Zn	−0.12	0.17	**0.42** ^***^	0.17	0.05	**0.49** ^***^	−0.08	**0.31** ^**^	

* Values in bold are significant at *p* < 0.05.

** Values in bold are significant at *p* < 0.01.

*** Values in bold are significant at *p* < 0.001.

### Path analysis

Direct and indirect effects of calcium, copper, potassium, magnesium, manganese, sodium, and zinc contents on iron content were estimated using path coefficient analysis and results were presented in Table 5. Manganese and phosphorus had high and positive direct effects on iron content. The high direct effect of manganese was accompanied with a significant and positive correlation. In contrast, the high direct effect of phosphorus and its negative indirect effect *via* calcium made the total correlation insignificant. Zinc had a considerable and positive direct effect as well as positive indirect effects *via* manganese and phosphorus along with a significant correlation. Calcium had moderate and positive direct effect but its negative and moderate indirect effect *via* phosphorus made the total correlation coefficient insignificant. Potassium had a negative and moderate direct effect, but its indirect and positive effect *via* phosphorus led to an insignificant correlation. Residual effect of 0.61 was observed, which showed that only 39% of the variability was explained by the eight minerals traits investigated in this study. Plus, 61% variability might be controlled by other contributing traits to iron content that were not included in the present study.

**Table 5:** The direct (bold diagonal numbers) and indirect effects of eight mineral elements on iron content in a population of 70 advanced lines of *G. gynandra*.

Minerals	Ca	Cu	K	Mg	Mn	Na	P	Zn	Fe
Ca	**0.26**	0.01	−0.03	0.06	0.07	0.00	−0.24	−0.02	0.11
Cu	−0.03	**−0.06**	−0.01	−0.02	−0.06	0.00	0.17	0.03	0.01
K	0.03	0.00	**−0.21**	0.00	0.01	0.02	0.10	0.03	−0.02
Mg	0.18	0.01	0.00	**0.09**	0.15	0.01	−0.16	0.01	0.28^*^
Mn	0.05	0.01	−0.01	0.03	**0.39**	0.01	−0.07	0.09	0.49^***^
Na	0.02	0.00	−0.06	0.01	0.05	**0.06**	−0.03	−0.01	0.03
P	−0.15	−0.03	−0.05	−0.03	−0.06	0.00	**0.42**	0.05	0.14
Zn	−0.03	−0.01	−0.04	0.00	0.19	0.00	0.13	**0.17**	0.42^***^

Residual effect = 0.61.

* Correlation value significant at *p* < 0.05.

*** Correlation values significant at *p* < 0.001.

### Clustering patterns among genotypes

**Figure 4:**
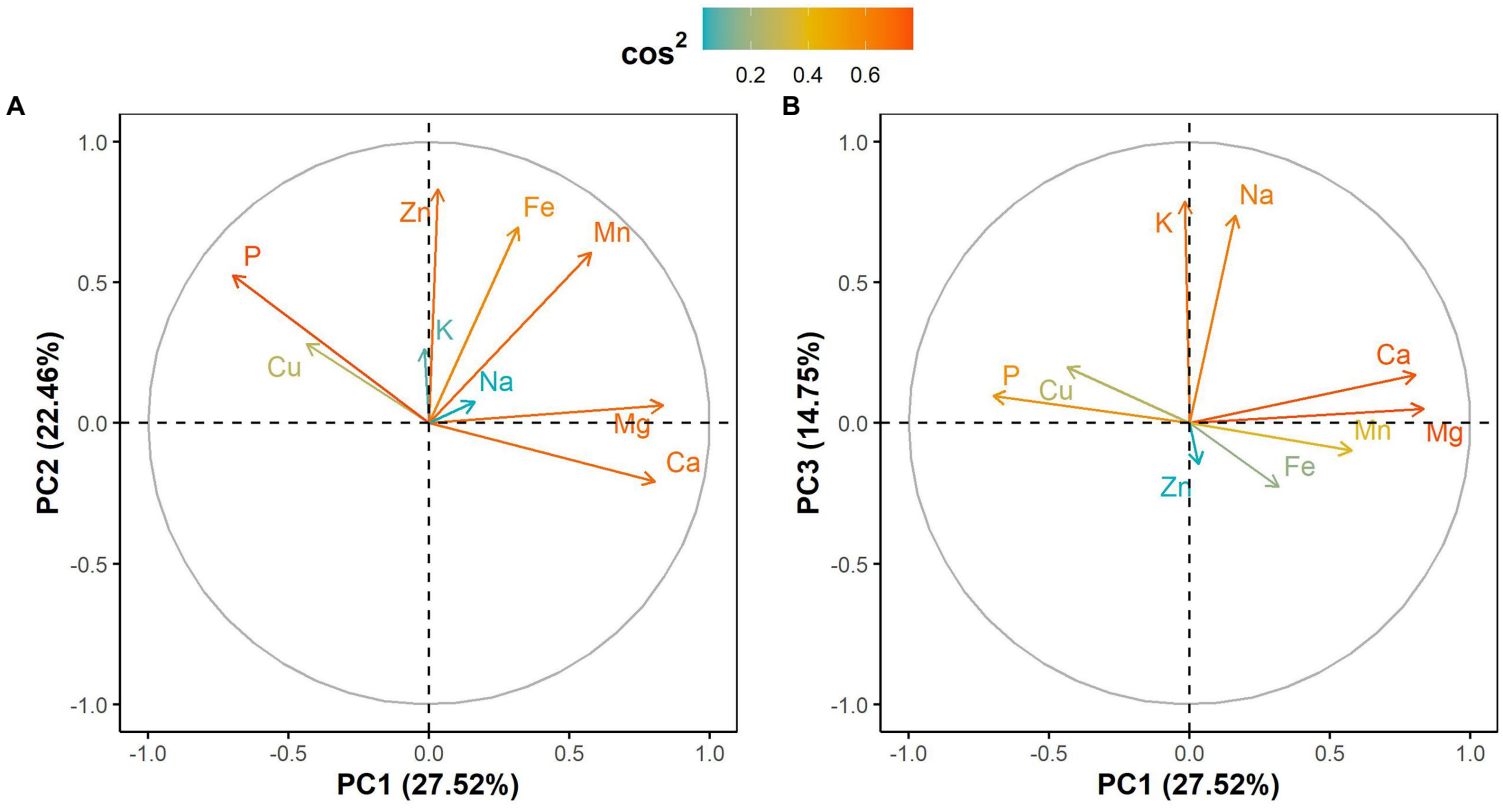
Correlation circle showing leaf mineral elements projection on **(A)** the first two principal components and **(B)** the first and third principal components. Cos^2^ refers to the quality of representation for variables on the principal component.

**Figure 5:**
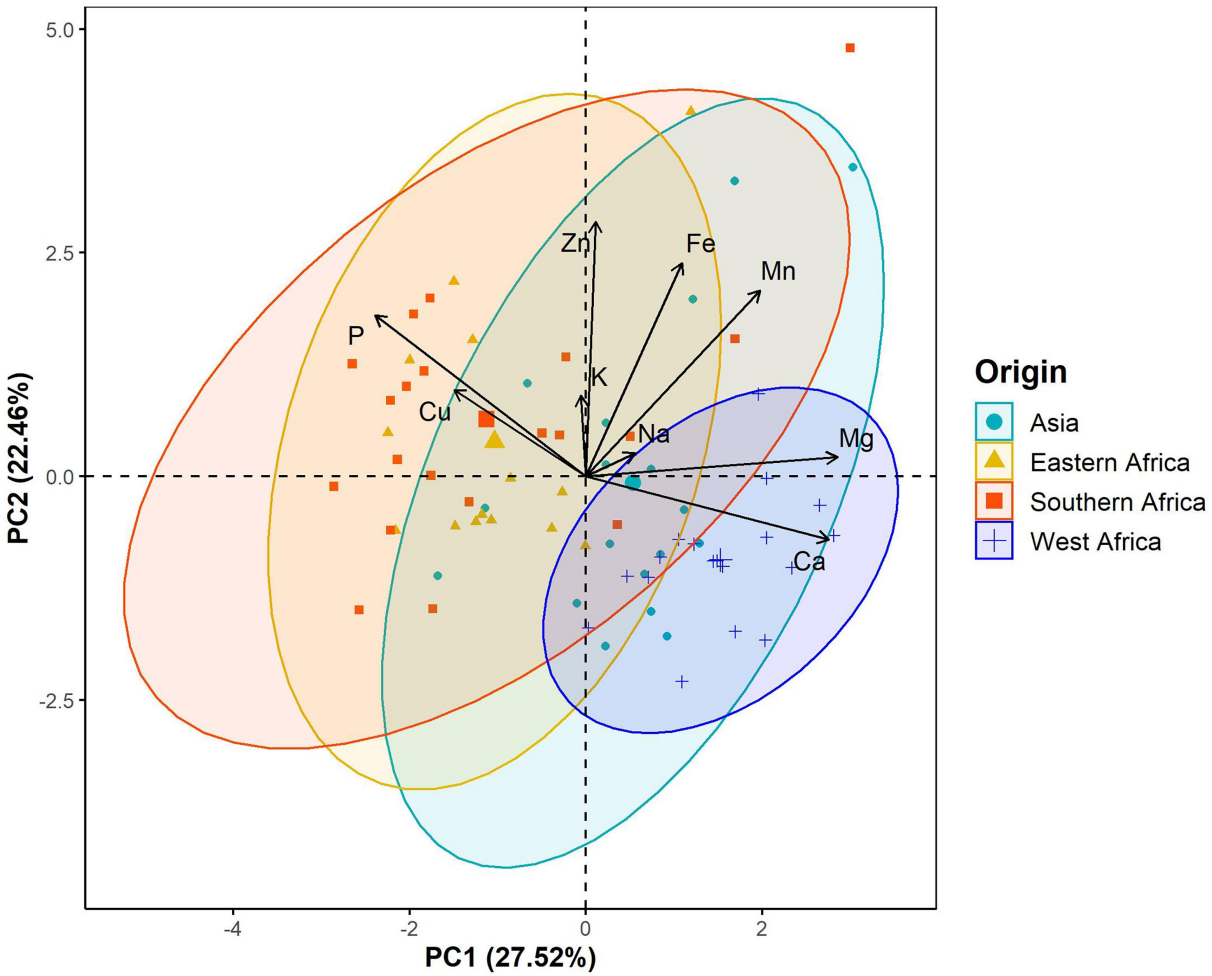
Biplots of the first principal component (PC1) versus the second principal component (PC2) for the leaf elemental composition in a population of 70 advanced lines of *G. gynandra*. Ninety percent bivariate ellipses were represented for lines from the same geographical origin. Asia (*n* = 18), Eastern Africa (*n* = 14), Southern Africa (*n* = 20); and West Africa (*n* = 18).

The principal component analysis (PCA) showed that the first three components explained 64.73% of the total variation in the leaf elemental composition (Figure 4; Supplementary Table 2). The first principal component retained 27.52% of the total variation and was positively and significantly correlated with calcium and magnesium contents but negatively with phosphorus (Figure 4; Supplementary Table 2). Iron, zinc and manganese concentrations were positively and significantly associated with the second principal component, which explained 22.46% of the total variation (Figure 4; Supplementary Table 2). The third principal component accounted for 14.75% of the total variation and was significantly and positively correlated with potassium and sodium contents (Figure 4; Supplementary Table 2). Furthermore, the PCA differentiated West African genotypes from both Eastern and Southern African genotypes, while Asian genotypes were spread between the West African and the Eastern and Southern African genotypes (Figure 5). The PCA biplot based on the first two components showed that the West African genotypes were characterized by calcium and magnesium contents. In contrast, Eastern and Southern African genotypes had substantial phosphorus and copper contents, with some genotypes having high iron, zinc and manganese contents (Figure 5).

**Figure 6:**
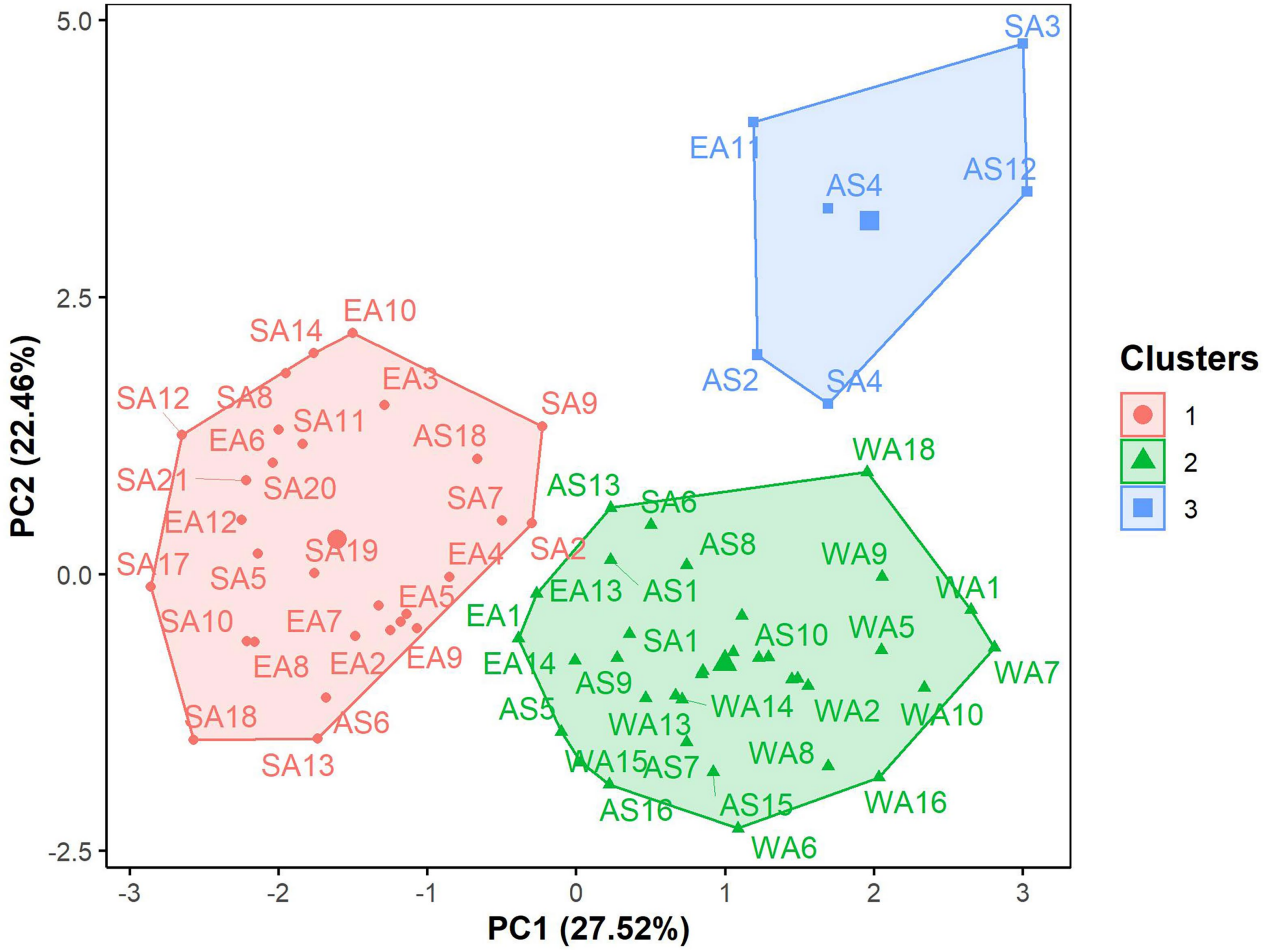
Factor map displaying the grouping pattern of 70 advanced lines of *G. gynandra* based on the hierarchical clustering on principal components analysis (HCPC). Cluster 1 (*n* = 31), Cluster 2 (*n* = 33) and Cluster 3 (*n* = 6). AS: Asia; EA, Eastern Africa; SA, Southern Africa; WA, West Africa.

**Figure 7:**
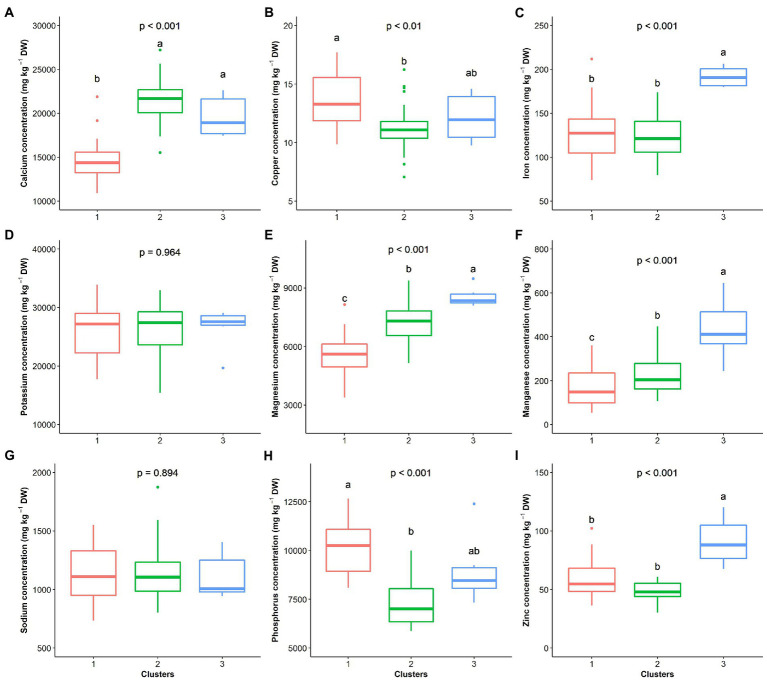
Clusters’ performance comparison based on nine elemental compositions of the leaf of *G. gynandra*. **(A)** Calcium content; **(B)** Copper content; **(C)** Iron content; **(D)** Potassium content; **(E)** Magnesium content; **(F)** Manganese content; **(G)** Sodium content; **(H)** Phosphorus content; and **(I)** Zinc content. Cluster 1 (*n* = 31), Cluster 2 (*n* = 33) and Cluster 3 (*n* = 6). Boxplots with the same alphabetic letter are not significantly different at *p* < 0.05 according to Dunn’s *post-hoc* test.

The hierarchical clustering on principal components classified the 70 *G. gynandra* genotypes into three clusters (Figure 6), whose characteristics are presented in Figure 7. Cluster 1 consisted of 41.43% (*n* = 29) of all genotypes and predominantly genotypes from Eastern (*n* = 10) and Southern (*n* = 16) Africa with three genotypes from Asia and therefore was named East/Southern African. Cluster 1 was characterized by low calcium, magnesium and manganese contents but had high phosphorus and copper contents with moderate iron and zinc contents. Genotypes in cluster 2 were mainly from West Africa (18) and Asia (12), with few from Southern (2) and Eastern (3) Africa (Figure 6). Cluster 2 encompassed 50.00% of all genotypes and was called Asian/West African. High calcium content together with moderate magnesium and manganese contents and low phosphorus, copper and zinc contents described cluster 2 (Figure 7). The last cluster, cluster 3, was composed of six genotypes, three, two, and one from Asia, Southern Africa and Eastern Africa, respectively. Cluster 3 was the best and was characterized by high iron, zinc, magnesium and manganese contents with moderate calcium content (Figure 7).

## Discussion

### Elemental status of *Gynandropsis gynandra* leaves

Leaves of *G. gynandra* are highly nutritious and rich in potassium, calcium, sodium, phosphorus, magnesium, iron, manganese, zinc and copper. This aligns with previous reports on the species leaf mineral content and potential in improving human nutrition (Jiménez-Aguilar and Grusak, 2015; Omondi et al., 2017; Moyo et al., 2018; Thovhogi et al., 2021). The concentrations in iron, zinc, calcium, magnesium, manganese, phosphorus, copper, potassium, and sodium were comparable with those reported by Moyo et al. (2018), Omondi et al. (2017) and Thovhogi et al. (2021), but with some differences. For instance, the highest iron content in the present study (430.72 mg kg^−1^) was comparable to that reported by Thovhogi et al. (2021) (431.3 mg kg^−1^) but significantly lower than that of Omondi et al. (2017) (5,892 mg kg^−1^) with the latter pointing out potential contamination of their samples by dust. The differences observed might also be associated with the genotype, environment and agricultural practices. Moyo et al. (2018) showed the superiority in mineral contents of *G. gynandra* over *Beta vulgaris* L. (Swiss chard) and *Brassica oleracea* var. *capitata* (cabbage), two world leading-consumed vegetables, with *G. gynandra* having 3.3- and 5.5-fold phosphorus, 1.4- and 1.8-fold potassium, 2.7- and 10.4-fold calcium, 4- and 2-fold iron and 1.2- and 2.1- fold zinc contents more than *B. vulgaris* and *B. oleracea var. capitata*, respectively. The calcium, magnesium, and potassium concentrations in the leaves of *G. gynandra* were higher than those reported in *Amaranthus* species (Shukla et al., 2006; Sarker and Oba, 2019).

Given the high mineral content, regular consumption of spider plant will be strategic in addressing micronutrient deficiencies and providing better human health because of the diverse essential biological, physiological, and metabolic functions of minerals in the human body. There are multiple roles of iron in the human body, which include: (i) serving as an oxygen carrier through red blood cell hemoglobin from the lungs to tissues, (ii) acting as an electron transporter within cells, and (iii) representing an essential component of enzyme machinery and DNA synthesis (Conrad and Umbreit, 2000; FAO and WHO, 2004). Iron represents one of the most deficient micronutrients in the diet of many populations, especially in Asia and Africa, and is responsible for diseases, including anemia, which mostly affects children, and pregnant and reproductive stage women in marginal regions of the world (Drakesmith et al., 2021). The recommended dietary allowance (RDA) for iron is between 7 and 18 mg/day, depending on age group (Institute of Medicine, 2006). With a serving size of 100 g fresh weight (FW), leaves of *G. gynandra* could provide 7.88 to 20.27% of the recommended dietary allowance (RDA) depending on the age group, with the lowest being for women between 19 and 50 years old (see Supplementary Table 3). This agreed with previous reports on the species (Van Jaarsveld et al., 2014; Jiménez-Aguilar and Grusak, 2015). Therefore, meeting the daily recommended intake of vegetables (300 g; Willett et al., 2019) using spider plant will contribute up to 50% of the RDA of iron. More importantly, a consumption of 300 g of leaves of genotypes in cluster 3 would provide 80% of RDA for adults, except premenopausal women. Spider plant would, therefore, contribute to alleviating iron deficiency.

Zinc is the second essential micromineral, and because of its ability to bind to several enzymes and transcription factors (Chasapis et al., 2020). Zinc deficiency remains an important health challenge in many low- and middle-income countries, including Sub-Saharan Africa (SSA), with a high prevalence in children, and pregnant and reproductive stage women, with consequences associated with increased child morbidity and mortality, adverse maternal health and pregnancy, and impaired childhood growth (Gupta et al., 2020). The consumption of 100 g fresh leaves of *G. gynandra* may provide between 5 and 7% of the RDA to adolescents and adults, respectively. This observation agreed with previous reports on the low contribution of the species and some other leafy vegetables to the diet for zinc (Gowele et al., 2019; Ejoh et al., 2021). However, spider plant could be a good source of zinc (19.84% of the RDA) for infants between 1 and 3 years old (see Supplementary Table 3).

Calcium confers rigidity to the skeleton (main cation of bone mineral), intervenes in most metabolic processes and is the second messenger of signals between the intracellular machinery and the plasma membrane (Bronner and Pansu, 1999; Power et al., 1999). Magnesium, mostly in muscles and soft tissues but low in extracellular fluid, acts as a cofactor of more than 300 enzymes involved in many physiological and biological processes (FAO and WHO, 2004; Glasdam et al., 2016). Depending on age group, the RDAs for calcium and magnesium were 500–1,300 mg/day and 80–430 mg/day, respectively (Institute of Medicine, 2006). While consuming 100 g of fresh leaves of spider plant provides 15% of RDA on average for adolescents, the same serving provides approximately 39 and 20% for infants (1–3 years old) and adults under 50 years old, respectively. In addition, *G. gynandra* leaves (100 g FW) could provide more than 50% of the RDA of magnesium for infants and children, with approximately 89% of the RDA to infants (1–3 years old; see Supplementary Table 3). Spider plant can be used to supplement calcium and magnesium for infants and children.

Manganese is a necessary nutrient for the human body, as it is crucial for the antioxidant system, development and metabolism (Avila et al., 2013). Manganese deficiency was associated with generalized growth impairment, birth defects, reduced fertility, impaired bone formation, altered metabolism of lipids, proteins and carbohydrates, and several diseases (e.g., Down’s syndrome, epilepsy, Perthest disease, osteoporosis Mseleni disease; Avila et al., 2013). Irrespective of the age group, a serving size of 100 g of fresh leaves of spider plant could significantly supply the daily requirements of manganese (>100%, see Supplementary Table 3). Spider plant is, therefore, a prime source of manganese.

Potassium constitutes the major intracellular cation in the human body and refers to an electrolyte due to its role as an electrical charge messenger that activates various nerve and cell functions (Sobotka et al., 2008). It is essential for the maintenance of normal levels of fluid inside cells. Potassium is also involved in building proteins and muscle, maintaining normal body growth, and controlling the electrical activity of the heart and the acid–base balance. Potassium also helps heart muscle contraction and supports normal blood pressure (Martínez-Ballesta et al., 2010). Sodium represents the major extracellular cation and is vital in regulating transmembrane gradients, fluid balance (maintaining normal fluid levels outside of cells), and blood pressure (Thomas and Bishop, 2013). Abnormal levels of potassium and sodium may lead to various pathological disorders, including hypernatremia, hyponatremia, hyperkalemia, hypokalemia (Ganong William, 2005; Pohl et al., 2013; Thomas and Bishop, 2013). Cardiac arrhythmia may result from a sudden loss of potassium (Pohl et al., 2013). The maintenance of the flux of these two electrolytes is assured by the Na^+^/K^+^-ATPase pump (Pivovarov et al., 2019). Ma et al. (2022) showed that higher sodium and lower potassium intakes were associated with a higher cardiovascular risk. Therefore, increasing potassium intake and reducing sodium intake is required, with a call for attention to the diet’s Na:K ratio (Baer et al., 2022). The RDAs of sodium and potassium were 1,000–1,500 mg/day and 2,000–3,400 mg/day, respectively (National Academies of Sciences Engineering and Medicine, 2019). A serving size of 100 g fresh leaves of *G. gynandra* may provide 0.81–1.22% of the RDA for sodium and 8.28–14.08% of the RDA for potassium according to the age group (see Supplementary Table 3). Interestingly, the positive correlation between sodium and potassium associated with the low sodium and high potassium content with a high K/Na ratio (K/Na = 23) of spider plant leaves is an important outcome and shows the potential of the species in addressing cardiovascular risk, blood pressure, maintaining electrolyte balance and muscular function. This agrees with previous reports on the richness of green leafy vegetables as a source of potassium (Ejoh et al., 2021).

Phosphorus is essential for many metabolic processes, particularly those involved in maintaining acid–base balance (Chongtham et al., 2021). Deficiency and excess of phosphorus are called hyperphosphatemia and hypophosphatemia, respectively. Phosphorus deficiency is unusual, but when it occurs, it is associated with painful bones, skin sensitivity, numbness, fatigue, anxiety, changes in body weight, irregular breathing and growth retardation (Renkema et al., 2008; Martínez-Ballesta et al., 2010). Given that the RDAs of adolescents and adults are 1,250 and 700 mg/day, respectively, consumption of 100 g of fresh leaves of spider plant could provide 7.30 and 13.05% of RDAs for adolescents and adults, respectively (see Supplementary Table 3).

Copper functions as a vital constituent of many metalloenzymes (monoamine oxidase, ferroxidases, diamine oxidase, dopamine b-monooxygenase), which act as oxidases in molecular oxygen reduction (Institute of Medicine, 2006). Copper deficiency is linked to diseases such as osteoporosis, hemosiderosis, abnormal bone formation with skeletal fragility, rheumatoid arthritis, hypochromic anemia, neutropenia, hair and skin hypopigmentation, lowered immunity, joint pain, vascular aberrations and kinky hair (Watts, 1989; Bhattacharya et al., 2016). The RDA of copper varies from 0.34 to 0.9 mg/day (Institute of Medicine, 2006). Therefore, the consumption of 100 g of fresh leaves of spider plant may provide between 14.42 and 38.19% of the daily requirement of copper. Specifically, a serving of recommended intake of vegetables (300 g) using spider plant may contribute up to more than 40% of the RDA for adolescents and adults (see Supplementary Table 3). Spider plant, would, therefore, contribute to alleviating copper deficiency.

Given the above role of each mineral in human health, the positive correlations observed between calcium and magnesium, magnesium and manganese, calcium and manganese, copper and phosphorus, iron and zinc, zinc and manganese, iron and manganese, sodium and potassium, potassium and phosphorus, phosphorus and zinc are of great importance in maintaining the proper functioning of the human body. The positive association between iron, zinc and manganese will reinforce the immune and antioxidant systems (Cannas et al., 2020). The association between calcium, magnesium and manganese is of importance in strengthening the bones, teeth, and nervous system (Quintaes and Diez-Garcia, 2015). More importantly, these positive associations show the potential contribution of spider plant leaves in maintaining blood pressure, preventing cardiovascular disease, improving enzyme machinery, energy metabolism, fluid-electrolyte balance, regulating cell volume, and cell signal transduction. In addition, the leaves could contribute to improving the anti-inflammatory system, muscle contraction and relaxation, reproductive system, nucleic acid and protein synthesis, gene expression regulation, cell cycle progression, apoptosis and homeostasis.

Therefore, introducing the spider plant into the human diet will provide key essential minerals to overcome hidden hunger, as the species is also a rich source of vitamins and important phytochemicals (Sogbohossou et al., 2019, 2020; Chataika et al., 2021; Moyo and Aremu, 2022). Based on the elemental status of *G. gynandra* leaves, the species can be used in biofortification programs, including medical supplementation and product fortification. Efforts are still needed to popularize and grow the species on large scale within and across countries/continents. To this end, genotypes in cluster 3 are potential candidates for species promotion. Several factors can affect leaf nutritional values, including soils, agronomic practices (fertilization, harvest time), developmental stages, cooking methods, and postharvest techniques. As nutritional value is genotype-specific, more investigations are needed to assess the effects of these factors on the nutritional values of the species (Moyo et al., 2018; Sogbohossou et al., 2019; Achigan-Dako et al., 2021). Other key components include the bioavailability of nutrients and the effects of the different cooking processes on bioavailability. Furthermore, the bioavailability of minerals depends on various factors, mainly concentration of anti-nutrients, such as phytic acid, oxalic acid, tannins and total polyphenols among others. Consequently, further studies should assess the variability in antinutrients (phytic acid, acid oxalic, etc.) among these advanced lines to establish the bioavailability profile of each genotype.

### Genetic variation of leaf elemental composition in *Gynandropsis gynandra*

Spider plant exhibits a significantly wide range of variations in the leaf elemental composition, representing an important resource for breeding programs. This confirms previous reports of significant variation in leaf mineral concentrations among genotypes of *G. gynandra* (Omondi et al., 2017; Thovhogi et al., 2021). The difference between these previous studies and the present study is the large collection used and the origin of the genotypes being from Asia and different regions of Africa, making our study more comprehensive, as most previous studies used genotypes from Eastern and Southern Africa (Jiménez-Aguilar and Grusak, 2015; Omondi et al., 2017; Thovhogi et al., 2021). Genotypes from this African region were found in the present study to cluster together and share the same genotypic background, as evidenced by Sogbohossou (2019). The observed variability was driven by the diverse provenance of the genotypes used as reported origin dependence in morphology (Wu et al., 2018; Sogbohossou et al., 2019), vitamin contents (Sogbohossou et al., 2019), secondary metabolite concentrations (Sogbohossou et al., 2020), seed mineral composition, seed morphology and germination (Blalogoe et al., 2020), antioxidant activity (Chataika et al., 2021), and photosynthesis traits (Reeves et al., 2018) in the species. The local adaptation in the species might further explain this.

### Differentiation of genotypes and evidence of local adaptation

The present study demonstrated three groups in *G. gynandra* based on the leaf elemental composition, including two major groups, the first being the East/Southern African group and the second being the Asian/West African group. The East/Southern African group is characterized by high phosphorus, copper and zinc contents, while the Asian/West African group had higher calcium, magnesium and manganese contents. This grouping was similar to that obtained by Reeves et al. (2018) based on DNA sequencing and phylogenetic reconstruction and photosynthesis traits of nine accessions from Asia, Eastern, Southern and West Africa. Furthermore, this difference might be associated with the role of minerals (phosphorus, copper, zinc, calcium, magnesium and manganese) in photosynthesis and many other physiological, biochemical and metabolic processes in plants, but also adaptability to the stress tolerance (Hänsch and Mendel, 2009; Maathuis, 2009). Another reason could be the induced changes by the environmental factors of the genotype origin with results of specific ion accumulation over time as local adaptation strategies (Huang and Salt, 2016). This signal of geographical association, particularly local adaptation, with the elemental composition has also been reported for the leaves of accessions of *Arabidopsis halleri* from different European ecological regions (Stein et al., 2017) and for fruits in Indian accessions of *Artocarpus heterophyllus* (Debbarma et al., 2021). The observed variation offers an opportunity to investigate genes associated with element or ion accumulation in the species as strategies for environmental adaptation, as reported in *Arabidopsis thaliana* (Campos et al., 2021). In-depth studies are required to understand the species’ ability to absorb nutrients from soils and to what extent soil affects the leaf elemental composition in the species. The local adaptation might further be explained by the genotype × year interaction variance greater than the genotypic variance, which might translate the phenotypic plasticity in the species, as QTLs by environmental interactions were observed to control mineral composition divergence in rice (Tan et al., 2020), maize (Asaro et al., 2016), and switchgrass (Zhang et al., 2021b). However, evaluation under different environmental conditions is required. Because this study focused on leaves, future studies should include different plant parts, including stems, flowers, pods, and seeds.

### Breeding and biofortification avenues for minerals-dense cultivars in *Gynandropsis gynandra*

Given the considerable variability observed, the present study offers several rooms for breeding nutrient-dense cultivars to tackle hidden hunger. The genotypes in cluster 3 are candidates for release and use in programs tackling micronutrient iron and zinc deficiencies. However, the biomass potential of the present germplasm used in this study should be assessed to identify morphological traits associated with mineral contents, as done by Sogbohossou et al. (2019), between morphology and vitamin concentrations in the species. Understanding the gene action controlling the leaf mineral content will play a key role in designing appropriate breeding strategies for improved cultivar development. In addition, genes controlling the leaf mineral content should be deciphered using a large natural collection and advanced populations, such as multiparent advanced generation intercross (MAGIC), recombinant inbred lines (RILs) and nested association mapping (NAM). These populations could be developed using the genotypes from clusters 1 and 2. Several methods could be used, including genome-wide association studies (GWAS) and QTL mapping, which will be easier with ongoing efforts to release the genome of *G. gynandra* (Hoang et al., 2022).

The positive correlation between iron and zinc offers the possibility for simultaneous selection, as low heritability was observed for iron but moderate for zinc. This correlation contrasted with that observed by Omondi et al. (2017) and Thovhogi et al. (2021), who found no association between iron and zinc. This might be associated with the germplasm used, and these authors used genotypes from the same geographical region, East/Southern Africa. Furthermore, the absence of correlation between iron content and all other minerals contents at the genotypic level revealed that the environment played a significant role in the concentration of iron in spider plant as well as its correlation with other traits. The positive correlation observed between calcium and magnesium, and copper and phosphorus was also reported by Thovhogi et al. (2021), offering the possibility for simultaneous selection. Therefore, calcium and copper contents could be increased with magnesium and phosphorus contents, respectively. Similarly, the positive correlation between the concentrations of magnesium and manganese as well between potassium and sodium at both phenotypic and genotypic levels showed that high magnesium and potassium content would be associated with high manganese and sodium content, respectively. Phosphorus had negative and significant correlations with calcium and magnesium contents at both phenotypic and genotypic levels, indicating that increased phosphorus content would decrease calcium and magnesium contents. Similarly, an increase in copper content would reduce magnesium, manganese, and sodium contents in spider plant as significant and negative correlations were observed between copper and these elements at genotypic level. As these correlations changed at the phenotype level with no correlation, therefore, environment influenced the association between copper content and magnesium, manganese, and sodium contents. At genotypic and phenotypic levels, the significant and positive correlation of zinc with manganese and phosphorus, revealed that zinc content directly increased with manganese and phosphorus contents. In contrast, zinc content would decrease magnesium and sodium contents at genotypic level, but this would be influenced by the environment as the absence of correlation at the phenotype level was observed between these traits. Consequently, manganese and phosphorus contents could be selected in improving zinc content in spider plant.

The high direct and positive effect along with a significant correlation of manganese content on iron content showed that manganese is the most contributing trait to iron content. Similarly, zinc had positive and considerable direct effect on iron content and a significant correlation. Consequently, direct selection based on manganese and zinc contents would significantly improve the iron content in spider plant. The high to moderate direct effect of calcium, potassium and phosphorus and their insignificant correlation with iron content indicated that direct selection based on these mineral elements would not be effective in improving iron content in spider plant. On the other hand, the high residual effect of the path coefficient analysis showed that not only leaf elemental composition contributed to iron content in spider plant. Therefore, there is a need to consider agro-morphological traits in future studies to decipher additional traits that could be used in improving iron content in the species.

The high genotype × year interaction variance observed in the present study shows the roles of both genotype and environment in the leaf elemental composition in *G. gynandra*. Further investigations should be conducted to estimate the extent of the influence of these components on the leaf mineral content in the species through multi-environmental trials. Moreover, the nutritional value of the leaves can be enhanced through biofortification as a complementary strategy and incorporated into the breeding strategy. Consequently, agronomic mineral biofortification (Buturi et al., 2021) could be achieved through the cultivation of *G. gynandra* in intensive agro-systems with the supply of nutrients through foliar fertilization, fertigation, soilless cultivation and organic fertilization. This will particularly contribute to increasing the levels of zinc and iron in spider plant leaves, as a positive association was observed between the two elements.

## Conclusion

The present study broadened the current knowledge on the nutritional value of *G. gynandra*, particularly its richness in minerals such as potassium, calcium, phosphorus, magnesium, iron, manganese, sodium, zinc, and copper. The species’ genetic variability in the leaf elemental composition was revealed and provided a strong basis for developing more ion-dense cultivars for improved nutrition. This variability displayed some signals of local adaptation to the origin of the genotype, with genotypes from Asia clustered together with West African genotypes, on the one hand, and those from Eastern Africa clustered together with Southern African genotypes, on the other hand, representing two significant gene pools for breeding higher nutritious cultivars. The Asian/West African group is rich in calcium, magnesium, and manganese, while the East/Southern African group had higher zinc, copper, and phosphorus contents. The two groups shared similar contents of iron, potassium and sodium. Additionally, genotypes (EA1, SA3, AS4, AS12, AS2, SA4) combining the characteristics of these two groups were identified in a different cluster and are a prime resource for large-scale promotion in programs/projects tackling micronutrient deficiencies. Leaves of *G. gynandra* can be used as a supplement and in food fortification. Due to the high mineral content of its leaves, *G. gynandra* is an important resource, which should be promoted and grown on large scale across the world as its introduction into diets could enhance the intake of mineral elements to combat hidden hunger. The significant genetic variation, moderate to high broad-sense heritability and high genetic gain for most mineral elements (zinc, calcium, phosphorus, potassium, magnesium, manganese sodium) contents, showed that selection would improve their concentration in leaves of spider plant. In contrast, environment was found to significantly influence copper and iron content in the species and that selection for copper and iron contents should consider the target environment. The positive correlation observed between calcium and magnesium, magnesium and manganese, copper and phosphorus, zinc and manganese, and zinc and phosphorus, offering the possibility for simultaneous selection. The high direct and positive effect along with a significant correlation of manganese and zinc content on iron content showed that direct selection based on manganese and zinc contents would significantly improve the iron contents in spider plant. Further investigations are required to assess micronutrient bioavailability, shelf-life and postharvest conditions, and cooking technique effects on the nutritional values of the species. Understanding the response to different growing conditions on leaf quality is required. Deciphering genes controlling each mineral and the extent of genotype-by-environment interaction effects on the leaf mineral composition is needed to boost more nutritious cultivars. Additionally, the potential association between morphological and leaf mineral contents should be assessed.

## Data availability statement

The original contributions presented in the study are included in the article/Supplementary material, further inquiries can be directed to the corresponding author.

## Author contributions

AH, EA-D, and JS conceived and designed the study. AH conducted the experiments, analyzed the data, and wrote the first draft. EA-D, ES, AO, MS, and JS critically revised the manuscript. All authors contributed to the article and approved the submitted version.

## Funding

This work was supported by the “Intra-Africa Academic Mobility Scheme” under project grant number 2016-2988 on “Enhancing training and research mobility for novel crops breeding in Africa (MoBreed)” funded by the Education, Audiovisual and Culture Executive Agency (EACEA) of the European Commission through a PhD scholarship awarded to Aristide Carlos Houdegbe. The scholarship was for academic training and research mobility and a research grant to complete a PhD degree at the University of KwaZulu-Natal (South Africa).

## Conflict of interest

The authors declare that the research was conducted in the absence of any commercial or financial relationships that could be construed as a potential conflict of interest.

## Publisher’s note

All claims expressed in this article are solely those of the authors and do not necessarily represent those of their affiliated organizations, or those of the publisher, the editors and the reviewers. Any product that may be evaluated in this article, or claim that may be made by its manufacturer, is not guaranteed or endorsed by the publisher.
