# Supplementary material for: Leaf elemental composition analysis in spider plant [Gynandropsis gynandra L. (Briq.)] differentiates three nutritional groups
Source: Front Plant Sci. 2022 Sep 2;13:841226. doi: 10.3389/fpls.2022.841226 (PMC9478508; doi:10.3389/fpls.2022.841226)
Supplement: Supplementary file 1 [file Table_1.DOCX]

Supplementary Table 1. Estimates of genetic parameters for the leaf minerals contents in 70 advanced lines of *G. gynandra* evaluated in 2020 and 2021.

| **Genetic parameters** | **Years** | **Ca** | **Cu** | **Fe** | **K** | **Mg** | **Mn** | **Na** | **P** | **Zn** |
| --- | --- | --- | --- | --- | --- | --- | --- | --- | --- | --- |
| $\sigma_{G}^{2}$ | 2020 | 17676314.7 ± 3229288.95 | 9.05 ± 2.31 | 1700.07 ± 422.82 | 16669271.65 ± 3643837.64 | 1525784.28 ± 265165.07 | 7661.03 ± 1307.43 | 37023.09 ± 6922.75 | 3581734.02 ± 616309.56 | 266.52 ± 46.73 |
|  | 2021 | 17177906.99 ± 3617136.09 | 2.87 ± 1.51 | 1721.47 ± 528.83 | 27107523.12 ± 6342627.56 | 3516612.67 ± 603905.04 | 32615.07 ± 5590.23 | 110735.06 ± 19204.59 | 4635441.8 ± 803749.83 | 589.96 ± 102.36 |
| $\sigma_{e}^{2}$ | 2020 | 2501538.88 ± 422837.25 | 7.18 ± 1.39 | 1354.65 ± 231.79 | 8605527.22 ± 1454599.59 | 62791.84 ± 10613.76 | 36.77 ± 6.26 | 6977.85 ± 1187.99 | 74773.68 ± 14476.08 | 15.64 ± 2.64 |
|  | 2021 | 7186883.22 ± 1306835.67 | 8.26 ± 1.61 | 2124.61 ± 374.27 | 17949555.64 ± 3074054.94 | 58527.93 ± 10111.75 | 426.63 ± 73.69 | 4001.03 ± 691.04 | 161962.67 ± 28201.32 | 22.09 ± 3.76 |
| $\sigma_{P}^{2}$ | 2020 | 18927084.14 ± 3440707.57 | 12.64 ± 3.00 | 2377.40 ± 538.72 | 20972035.26 ± 4371137.44 | 1557180.2 ± 270471.95 | 7679.41 ± 1310.56 | 40512.01 ± 7516.75 | 3619120.86 ± 623547.60 | 274.34 ± 48.05 |
|  | 2021 | 20771348.59 ± 4270553.93 | 7.00 ± 2.31 | 2783.77 ± 715.97 | 36082300.94 ± 7879655.03 | 3545876.63 ± 608960.91 | 32828.39 ± 5627.08 | 112735.58 ± 19550.11 | 4716423.13 ± 817850.49 | 601.01 ± 104.24 |
| $H^{2}$ | 2020 | 0.93 | 0.72 | 0.72 | 0.79 | 0.98 | 0.99 | 0.91 | 0.99 | 0.97 |
|  | 2021 | 0.83 | 0.41 | 0.62 | 0.75 | 0.99 | 0.99 | 0.98 | 0.98 | 0.98 |
| Mean (mg kg^-1^ dry weight) | 2020 | 17553.63 | 12.84 | 128.33 | 26859.04 | 5848.99 | 183.40 | 1139.84 | 8662.85 | 60.66 |
|  | 2021 | 19525.86 | 11.64 | 139.87 | 25909.06 | 7584.39 | 249.88 | 1146.99 | 8472.52 | 51.12 |
| GCV (%) | 2020 | 23.95 | 23.42 | 32.13 | 15.20 | 21.12 | 47.73 | 16.88 | 21.85 | 26.91 |
|  | 2021 | 21.23 | 14.55 | 29.66 | 20.10 | 24.73 | 72.27 | 29.01 | 25.41 | 47.51 |
| PCV (%) | 2020 | 24.78 | 27.69 | 37.99 | 17.05 | 21.33 | 47.78 | 17.66 | 21.96 | 27.30 |
|  | 2021 | 23.34 | 22.73 | 37.72 | 23.18 | 24.83 | 72.51 | 29.27 | 25.63 | 47.95 |
| ECV (%) | 2020 | 9.01 | 20.87 | 28.68 | 10.92 | 4.28 | 3.31 | 7.33 | 3.16 | 6.52 |
|  | 2021 | 13.73 | 24.69 | 32.95 | 16.35 | 3.19 | 8.27 | 5.51 | 4.75 | 9.19 |
| GA | 2020 | 8369.84 | 5.24 | 71.83 | 7498.31 | 2518.78 | 180.09 | 378.92 | 3878.46 | 33.15 |
|  | 2021 | 7764.35 | 2.23 | 67.21 | 9296.30 | 3847.07 | 370.82 | 679.39 | 4396.95 | 49.57 |
| GAM (%) | 2020 | 47.68 | 40.83 | 55.97 | 27.92 | 43.06 | 98.20 | 33.24 | 44.77 | 54.64 |
|  | 2021 | 39.76 | 19.20 | 48.05 | 35.88 | 50.72 | 148.40 | 59.23 | 51.90 | 96.97 |

$\sigma_{G}^{2}$, genotypic variance; $\sigma_{e}^{2}$, residual variance; $\sigma_{P}^{2}$, phenotypic variance; $H^{2}$, broad-sense heritability; GCV, genotypic coefficient of variation; PCV, phenotypic coefficient of variation; ECV, residual coefficient of variation; GA, genetic advance; GAM, genetic advance over mean.
