# Supplementary material for: Leaf elemental composition analysis in spider plant [Gynandropsis gynandra L. (Briq.)] differentiates three nutritional groups
Source: Front Plant Sci. 2022 Sep 2;13:841226. doi: 10.3389/fpls.2022.841226 (PMC9478508; doi:10.3389/fpls.2022.841226)
Supplement: Supplementary file 2 [file Table_2.DOCX]

Supplementary Table 2. Correlation between leaf mineral elements and the three first principal components.

| **Minerals** | **PC1 (27.52%)** | **PC2 (22.46%)** | **PC3 (****14.75%)** |
| --- | --- | --- | --- |
| Ca | **0.804***** | -0.207 | 0.171 |
| Cu | -0.436 | 0.282 | 0.198 |
| Fe | 0.317 | **0.696***** | -0.226 |
| K | -0.016 | 0.264 | **0.787***** |
| Mg | **0.832***** | 0.062 | 0.050 |
| Mn | 0.576 | **0.607***** | -0.098 |
| Na | 0.162 | 0.075 | **0.738***** |
| P | **-0.699***** | 0.526 | 0.096 |
| Zn | 0.032 | **0.831***** | -0.145 |

Values in bold indicated significant correlation at p < 0.001 (***).
