# Supplementary material for: Leaf elemental composition analysis in spider plant [Gynandropsis gynandra L. (Briq.)] differentiates three nutritional groups
Source: Front Plant Sci. 2022 Sep 2;13:841226. doi: 10.3389/fpls.2022.841226 (PMC9478508; doi:10.3389/fpls.2022.841226)
Supplement: Supplementary file 4 [file Data_Sheet_1.PDF]

Supplementary Figure 1

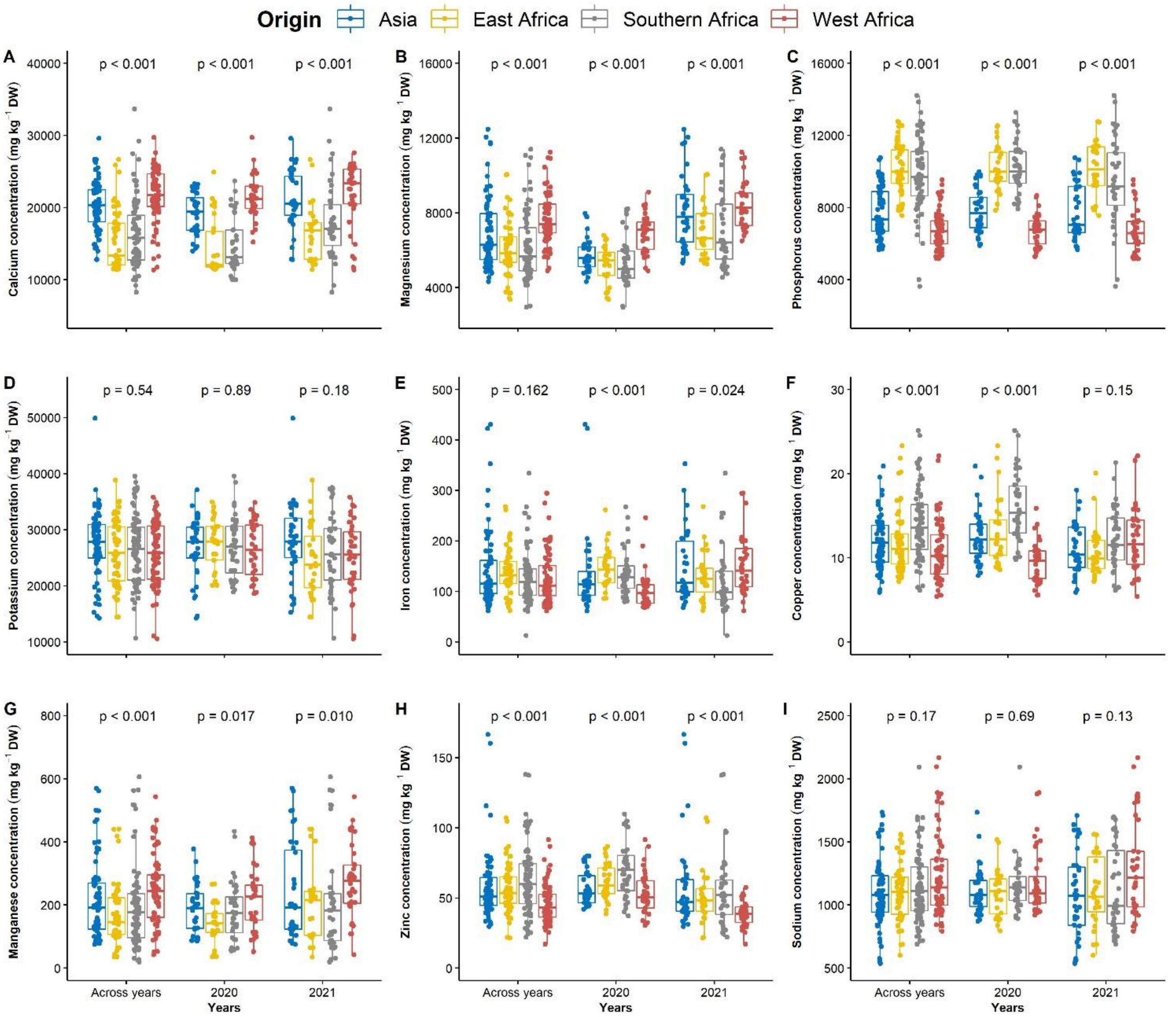

Supplementary Figure 1. Boxplots showing variation in nine leaf mineral contents among regions of origin of a population of 70 advanced lines of *G. gynandra* evaluated in 2020 and 2021. (**A**) Calcium content; (**B**) Magnesium content; (**C**) Phosphorus content; (**D**) Potassium content; (**E**) Iron content; (**F**) Copper content; (**G**) Manganese content; (**H**) Zinc content; (**I**) Sodium content. Asia (n = 18), East Africa (n = 14), Southern Africa (n = 20); and West Africa (n = 18).
